# Effect of a psycho-educational intervention for family members on caregiver burdens and psychiatric symptoms in patients with schizophrenia in Shiraz, Iran

**DOI:** 10.1186/1471-244X-12-48

**Published:** 2012-05-25

**Authors:** Farkhondeh Sharif, Maryam Shaygan, Arash Mani

**Affiliations:** 1 Department of Mental Health & Psychiatric Nursing, Community Based Nursing &Midwifery Research Center, Faculty of Nursing and Midwifery, Shiraz University of Medical Sciences, Shiraz, Iran; 2 Department of Mental Health Nursing, Faculty of Nursing and Midwifery, Shiraz University of Medical Sciences, Shiraz, Iran; 3 University Counseling Center, Shiraz University of Medical Sciences, Shiraz, Iran

## Abstract

**Background:**

This study explored the effectiveness of family psycho-education in reducing patients’ symptoms and on family caregiver burden.

**Methods:**

Seventy Iranian outpatients with a diagnosis of schizophrenia disorder and their caregivers were randomly allocated to the experimental (n = 35) or control groups (n = 35). Patients in the experimental group received antipsychotic drug treatment and a psycho-educational program was arranged for their caregivers. The psycho-educational program consisted of ten 90-min sessions held during five weeks (two session in each week). Each caregiver attended 10 sessions (in five weeks) At baseline, immediately after intervention, and one month later. Validated tools were used to assess patients’ clinical status and caregiver burden.

**Results:**

Compared with the control group, the case group showed significantly reduced symptom severity and caregiver burden both immediately after intervention and one month later.

**Conclusions:**

These results suggest that even need based short-term psycho-educational intervention for family members of Iranian patients with schizophrenic disorder may improve the outcomes of patients and their families.

**Trial registration:**

IRCT Number:138809122812 N1`

## Background

Schizophrenia is a severe mental illness, which is stressful not only for the patients but also for their family members. Between 50% and 80% of patients with schizophrenia live with or have regular contact with family members [1], and rely on relatives for housing, and emotional and financial support. Therefore, the quality of their relationships greatly influences the patients’ outcomes [2]. However, these families report high levels of burden related to caring for a member with schizophrenia [3,4]. As a result, studies have attempted to find a link between patients’ symptoms and family burden [5,6]. Patient stressors such as negative and disruptive symptoms have been linked to increased burden in caregivers of patients with schizophrenia. Winefield and Harvey (1993) found a significant positive correlation between the level of behavioral disturbance in the patient and caregivers’ distress [5]. However, one study found no link between these patient stressors and family burden [7].

There are different types of family interventions for reducing the patient/caregiver burden such as behavioral family management, psychoeducational family intervention, family therapy, etc. In a recent study that compared different models of family interventions, the researchers concluded that psychoeducation was more useful in decreasing the burden in the mothers caring for their child suffering from schizophrenia [8]. Recent changes in the treatment of schizophrenia disorder make use of both traditional and psycho-educational interventions to treat the symptoms [9-14]. Several other studies have also demonstrated the efficacy of family psycho-educational interventions in reducing of relapses, re-hospitalization [15-17] and family burden [18-20]. The psycho-educational intervention is a set of systematic intervention based on supportive and cognitive behavior therapy approach with emphasis on patients and family needs. The intervention is focused on increasing patient and family knowledge about disease, better adjustment to illness, communication and facilitating problem solving skills [21]. Despite the current emphasis on community-based care and family psycho-education for these patients [2,22,23], these approaches have not been attempted in Iran yet. In a study by Mottaghipour and her colleagues, Sutherland Mental Health Service in New South Wales was used to show that organizational changes are needed to implement a “family friendly service” [24]. In an Iranian study, Malakouti and his colleagues conducted a comparative study of the clinical outcomes of mental health workers and consumers’ family members as case managers with 12 months of home-visit services for 129 patients with Schizophrenia. Burden, knowledge, quality of life and the general health condition of the care-givers, as well as positive/negative symptoms and social skills of the consumers were evaluated. Most clinical variables were improved without significant differences between groups. The hospitalization rate was reduced by 67% [25].

In addition, there are only limited studies focusing on Iranian populations with focus on patients and family need assessment, and it is unclear whether family psycho-education, which has been recognized as effective in European and American populations, can be applied successfully in Iranian family. Therefore, it is important to test the efficacy of psycho-education in enhancing family knowledge about the illness and the ability to cope with care giving role in Iranian families with a member who has schizophrenia.

Iranian families are characterized by their intimate interpersonal relationships and many interactions among family members. Therefore, illnesses in one family member results in a substantial burden for the whole family. In addition, Iranian families report a low level of formal support services as compared with their Western peers [26].

Currently, there are no community mental health centers specifically for following up patients with schizophrenia in Iran. The patients mainly refer to psychiatrists or Psychiatric centers or primary healthcare centers that do not clearly address the specific needs of each family. Moreover, since mental illness is considered as a taboo in our cultural settings and many families are not aware of the needs and illness of their patients, they experience a great amount of burden. Also, the patients nor their families do not receive routine non-pharmaceutical treatment such as family interventions. Moreover, we do not have trained professionals in this regard to perform such interventions.

Considering the lack of routine long term psychoeducational programs for patients with schizophrenia and their families based on their specific needs, we aimed to investigate the efficacy of family psycho-education in reducing patients’ symptoms and its efficacy of family psycho-education in reducing family caregiver burden.

## Methods

### Design

This randomized controlled trial was conducted in Shiraz, a city of about 2.5 million inhabitants in southern Iran. Seventy caregivers patients with schizophrenia whose records were available at three psychiatric centers in Shiraz were randomized blindly to two groups considering the inclusion criteria and consulting with their psychiatrists. We developed our intervention based on the families’ needs and the modified existing literature in this regard [27,28]. The psycho-educational program consisted of ten 90-min sessions held during five weeks (two sessions each week). Each caregiver attended 10 sessions (in five week) on the afternoon of their choice from the point of suitability of time. Four psycho-educational groups of eight or nine caregivers each were arranged with the same contents, and the program was conducted by a psychiatric nurse or psychiatrist.

The goals and contents of each of the ten sessions are summarized in Table 1. The beginning of the first session was practically a needs assessment session in which we asked the caregivers about the types of issues and problems they have with their patients and what they would like to know about their patient’s condition in order to better organize the interventions. After doing subjects’ need assessment,each psycho-educational session included a variety of educational techniques designed to enhance the participant's learning and maintain their attention (for example; visual aids such as charts, film presentation and Microsoft PowerPoint slideshows). The first part of each session consisted of a lecture given by a psychiatrist or psychiatric nurse and the last part of each session (30 min) consisted of a question-and-answer and group discussion period. During this period, the caregivers described situations and incidents related to their patients and discussed alternative ways of coping with and resolving their difficulties with care giving. At the second session, the guest speaker was a patient with a DSM-IV diagnosis of schizophrenia who had a clinically stable status. He related his experiences with his illness and offered insights to the caregivers. At the end of the sessions, an educational booklet was given to all family members.

**Table 1:** Content of psycho educational program

**Session**	**Goals**	**Content**
1	To orient caregivers to the program and to create a trusting relationship between caregivers and instructors	· Assessment of family needs.
		· Overview of the program and introduction of instructors and members to each other.
		· Discussion of the importance of orientation to patient behaviors and symptoms
		· Completion of Brief Psychiatric Rating Scale and Family Burden scale by participants.
2	To understand schizophrenia, its symptoms and treatments, and its effects on patients and families	· Presentation of a patient with a DSM-IV diagnosis of schizophrenia with clinically stable status. The patient describes his experiences and offer insights. The instructor offers explanations of the symptoms and behaviors, and of their effects on the family.
		· Discussion of the etiology and treatments
		· Question-answer and group discussion.
3	To recognize the effect of medications and compliance.	· A review of the previous session.
		· Discussion of positive and negative effects of antipsychotic drugs and problems related to side effects.
		· Emphasis on the importance of drug compliance and maintenance.
		· Question-answer and group discussion.
4	To orient caregivers to the warning signs of relapse and relapse prevention	· A review of the previous session.
		· Discussion about warning signs of relapse.
		· Explanation of the family role in relapse prevention.
		· Exploration of family intervention when the relapse has occurred.
		· Question-answer and group discussion.
5-6	To improve communication skills in the family	· A review of the previous session.
		· Discussion of the importance of effective communication skills in the family and the role of environmental stress as a risk factor for schizophrenia relapse.
		· Discussion of skills for effective communication between family members.
		· Question-answer and group discussions.
7	To manage the patient’s symptom and skills in coping with them	· A review of the previous session.
		· Discussion of effective communication skills with patients when they have symptoms.
		· Discussion of token economy and negative reinforcement for managing patients’ symptoms.
		· Explanation of skills for coping with some of the patients’ symptoms.
		· Question-answer and group discussion.
8	To understand effective way to express emotion	· A review of the previous session.
		· Exploring intense emotions towards the patient.
		· Discussion of expressing emotion and emotional environment in the family.
		· Discussion of how to cope with the patient’s negative emotions.
		· Question-answer and group discussion.
9	To orient caregivers to stress management in the family	· A review of the previous session.
		· Introduction of the importance of stress management in the family.
		· Discussion of ways to reduce stress.
		· Question-answer and group discussion.
10	To orient caregivers to relaxation methods	· A review and summary of the contents of past sessions.
		· Practicing relaxation methods during sessions.
		· Conclusion.

Approval for the study was obtained from the Ethics Committee of Shiraz University of Medical Sciences. Written consent was obtained from the patients and their families. All participants were informed about the purpose of the study and about their right to withdraw at any time, and were assured that all personal information would remain confidential.

### Participants

The reviewed the records of all patients with schizophrenia which were available at the Psychiatric centers. We called those patients who met our inclusion criteria and told their family about their study and invited the patient and one of their family members who is the main caregiver to participate in our trial. The patients had lived with their families for at least two years. Therefore, seventy caregivers of outpatients with a diagnosis of schizophrenia disorder who were members of their immediate family were randomly allocated to the experimental (n = 35) or control group (n = 35). In the control group, only the patients received routine care (antipsychotic drug treatment) whereas in the experimental group, the caregivers participated in a psycho-educational program while the patients received antipsychotic drug treatment. The intervention was conducted by the same psychiatrist and psychiatric nurse. The co-researcher (assessor) was blind to study treatment and condition and completed the scales. We included caregivers whose patients had the following criteria: i) a diagnosis within the preceding 5 years of schizophrenia disorder according to DSM-IV criteria, and ii) no other Axis 1 disorder during recruitment. All caregivers who were invited to participate identified themselves as the primary caregiver with the greatest responsibility for providing care within the family, and they themselves had no known mental illness. It is necessary to mention that after the intervention a certificate of attendance was given to participant and thanked them accordingly.

The exclusion criteria for the study were: i) caregivers who had participated in another psycho educational program during the preceding year, ii) caring for more than one family member with mental illness, and iii) substance abuse problem in the patient.

### Instruments

Data were collected with a personal information sheet, the Iranian version of the Brief Psychiatric Rating Scale (BPRS) [29] which was completed for every patient by caregiver, and the Family Burden questionnaire [25]. The concept of family burden consists of two aspects, objective burden related to the performance of daily assistance activities, financial impact, behavior supervision and disruption of family routine, and subjective burden concerning worries about the patients and feelings of being disturbed by care giving activities.(26). A specially designed questionnaire was used to collect data on the participants’ age, sex, education, marital status, type of medication, the degree of patient compliance to taking medication, employment status, economic status, and relationship between the caregiver and the patient.

Before, immediately after and one month after the intervention, psychiatric symptoms were assessed with the BPRS. The BPRS is a widely applied instrument consisting of 16 symptom constructs for evaluating the psychiatric status of a patient and it is in four subscales: positive symptoms, negative symptoms, mania-hostility symptoms and depressive-anxiety symptoms. Each item is rated from 1 (absent) to 7 (very severe) (16 symptom constructs) [30]. This scale has been translated in persian and utilized in Fallahi’s (2007, 2011) studies. The coefficient for reliability of the tool was determined by chronbach’s alpha to be r = 0.8 in some studies [29,31,32]. Also Khodabakhshi Koolaee [2007] indicated satisfactory content validity and internal consistency with Cornbach’s alpha to be 0.72 [33].

The caregiver burden was estimated with the validated Persian version of the family Burden questionnaire. This instrument contains ten closed questions. It has been used in Iran and its reliability and validity has been proved by several studies. The reliability of the questionnaire was assessed by the Spearman-Brown correlation coefficient and reported to be 0.80 [26,33,34]. Also Schene reported the Cronbach’s alpha coefficient for reliability of the tool is based on internal consistency of 0.85 [35].

### Statistical analysis

SPSS v. 15 was used for the statistical analysis. At baseline, sociodemographic characteristics in the two groups were compared with the chi-squared test. Between-group comparisons of the variables were done with Student’s *t*-test and repeated measurement analyses of variance were used to determine whether the improvements in these variables were changed over time.

## Results

A total of 65 families completed the study. Five participants (two from the experimental group and three from the control group) dropped out before completion of the study for different reasons unrelated to the study. The two groups of patients and their families did not differ significantly in any of the sociodemographic characteristics. Mean age of the patients in the experimental group was 32.5 years and that of their caregivers in the same group was 50.5 years. Mean age of the patients in the control group was 30 years and that of their caregiver in the same group was 52.5. Women made up 63% of the patients in the experimental group and 43% in the control group. Most of the patients in both groups were single and unemployed. The majority of caregivers in both groups were mothers of patients, most of whom had primary education and belonged to the middle class. All of the patients were on antipsychotic medication. No patient in either group was hospitalized during the study period. There were no significant differences regarding demographic data between the groups.

**Figure 1:**
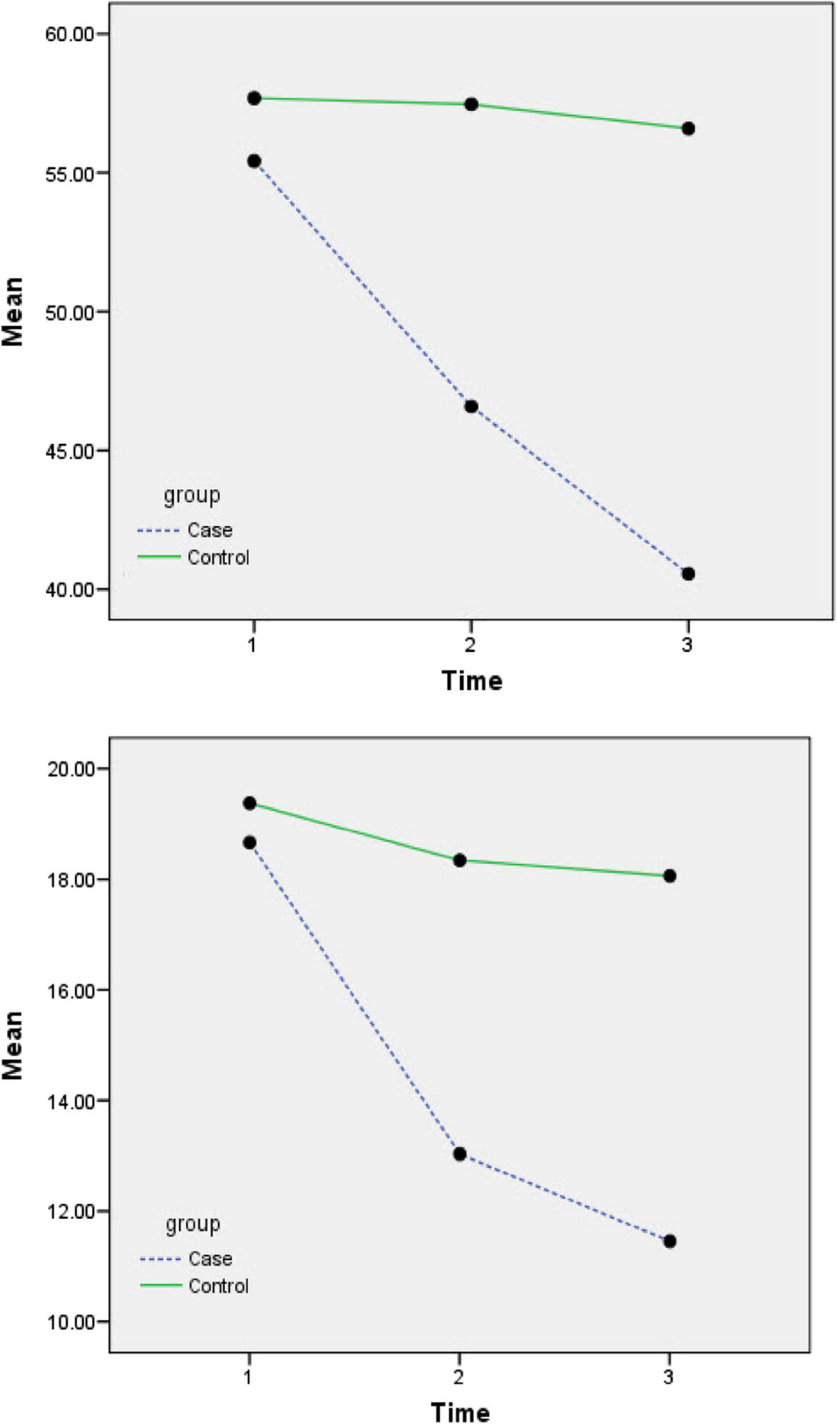
BPRS Means profile in experimental & control Group.

**Figure 2:**
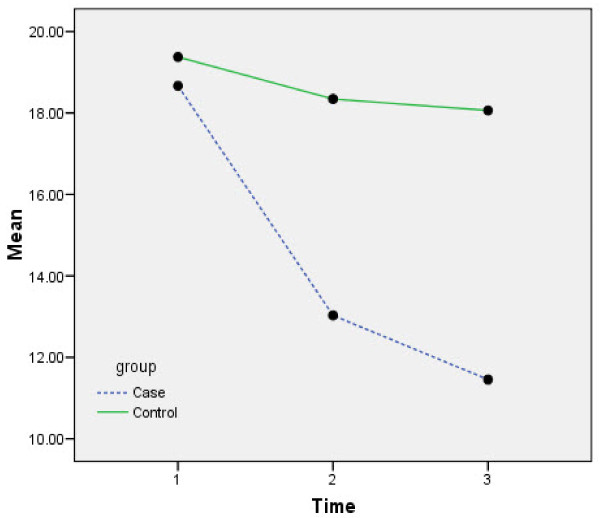
FBS Means profile in experimental & control Group.

The patients’BPRS Means profile in experimental & control Group is shown in Figure 1 and FBS mean profiles in experimental & control Group is shown in Figure 2.

The patients’ clinical status and family caregiver burden time 0 (baseline), time 1 (immediately after the intervention), and time 2 (one month post-intervention) is shown in Table 2.

**Table 2:** Patients’ clinical status and family caregiver time 0 (baseline), time 1 (immediately after the intervention), and time 2 (one month post-intervention)

**Variable**	**Experimental group (N=33)Mean**	**Control group (N=32) Mean**	**F**	**P <**
**BPRS global score**				
Time 0	55.42 ± 16.63	57.68 ± 20.36		
Time 1	46.57 ± 17.78	57.46 ± 20.90		
Time 2	40.54 ± 16.57	56.59 ± 20.19		
Effect of time			45.09	< 0.001
Interaction of time and treatment			34.08	< 0.001
Treatment			4.55	0.037
**BPRS positive**				
Time 0	2.96 ± 1.60	3.25 ± 1.73		
Time 1	2.64 ± 1.47	3.23 ± 1.77		
Time 2	2.17 ± 1.12	3.17 ± 1.72		
Effect of time			15.11	< 0.001
Interaction of time and treatment			10.09	< 0.001
Treatment			2.65	0.108
**BPRS negative**				
Time 0	3.81 ± 1.52	4.13 ± 1.64		
Time 1	3.15 ± 1.59	4.18 ± 1.60		
Time 2	2.87 ± 1.55	4.20 ± 1.62		
Effect of time			18.13	< 0.001
Interaction of time and treatment			24.76	< 0.001
Treatment			5.38	0.024
**BPRS manic/hostility**				
Time 0	2.72 ± 1.55	2.87 ± 2.00		
Time 1	2.54 ± 1.49	2.87 ± 2.05		
Time 2	2.17 ± 1.33	2.82 ± 2.03		
Effect of time			14.31	< 0.001
Interaction of time and treatment			9.56	< 0.001
Treatment			0.755	0.388
**BPRS depression/anxiety**				
Time 0	4.78 ± 1.69	4.96 ± 1.66		
Time 1	3.68 ± 1.69	4.89 ± 1.68		
Time 2	3.13 ± 1.75	4.78 ± 1.59		
Effect of time			38.46	< 0.001
Interaction of time and treatment			24.28	< 0.001
Treatment			6.38	0.014
**Family Burden**				
Time 0	18.66 ± 6.59	19.37 ± 6.22		
Time 1	13.03 ± 5.74	18.34 ± 6.5		
Time 2	11.45 ± 5.52	18.06 ± 6.68		
Effect of time			94.24	< 0.001
Interaction of time and treatment			45.08	< 0.001
Treatment			7.88	0.007

Comparisons of the baseline scores of the variables (positive symptoms, negative symptoms, mania-hostility symptoms, depressive-anxiety symptoms, global BPRS score and family burden) detected no significant differences between the two groups.

The findings after completion of the psycho educational program indicated statistically significant differences between the two groups for negative symptoms (especially uncooperativeness) and depressive-anxiety symptoms, an improvement in the global BPRS score, and a reduction in the family burden score, with respect to the baseline (Table 2). One month post-intervention, there were statistically significant differences between the two groups in negative symptoms (especially uncooperativeness), depressive-anxiety symptoms and positive symptoms. In addition, we found major improvements in the global BPRS score as well as a greater reduction in family burden score with respect to the baseline. The mean scores at time 0 (baseline) and time 2 (one month post-intervention) indicated that the experimental group had improved steadily in the global BPRS score (P < 0.037) and family burden (P < 0.0001).

## Discussion

The family psychoeducation in this study demonstrated positive effects in reduction of family burden and patients symptoms immediately and one month after the intervention. Most previous family psychoeducational studies have focused on European and American populations [35], whereas some studies have been carried out in Asian populations including the Iranian population. Nevertheless, Iranian families are characterized by their intimate interpersonal relationships and many interactions among family members. Therefore, illnesses in one family member results in a substantial burden for the whole family. In addition, some Western studies reported formal support services for their patients [26,36]. The present study focused on the impact of psycho educational intervention in Iranian families in which one member has schizophrenia. The results of our psycho educational intervention were encouraging and the caregivers in the experimental group indicated a significant decrease in family burden. Also, there was an improvement in most aspects of the BPRS in the patients they took care of. Improvement in the patient’s clinical status and decreases in family burden may be related to the family’s awareness of strategies for dealing with daily problematic situations [37]. In addition, our results may be related to family orientation to the patient’s symptoms and behavior, and to their skills of coping with them, consistent with other studies [38]. As a result of our intervention, family members may have learned to understand effective ways of expressing emotions in the family context. Also Xiong and her colleagues in their study about family-based intervention for schizophrenic patients in china mentioned that improvements in patients’ symptoms may have been related to enhanced treatment compliance because families were better able to supervise the patient’s use of antipsychotic drugs [36]. Also Niksalehi and colleagues (2011) reported that nursing home care services were more effective than telephone follow-ups for schizophrenic mental conditions [32].

The findings of this study are consistent with those of earlier research in other countries suggesting that participation in an educational and supportive group for caregivers of patients with schizophrenia results in better acceptance of the illness, and enhanced adaptability to their care giving role [38]. Medvene and Krauss found that mutual aid groups for caregivers of the mentally ill resulted in increased comfort in talking with other caregivers about their problems in care giving situations [39]. In addition, several studies have reported that the interactions between caregivers in groups may give rise to emotional support and practical help, which is extended to the post-intervention period [40]. Family psycho educational intervention may have a positive effect on family burden by reducing many patient risk factors of burden. This is consistent with the positive therapeutic effects of psycho education on family burden reported by other authors [41,42]. Also Reza and colleagues (2004) in their study indicated that psycheducational programs can facilitate social adjustment of Iranian psychiatric patients [43].

Our control group, which received routine care, showed little improvement in the patient’s clinical status and family burden. These results may reflect the fact that routine services for schizophrenia patients and their families in Iran do not meet the patients’ and families’ needs.

Our study had some limitations. The sample size was relatively small, so larger studies are needed to confirm these results. The improvements in the patients’ symptoms and family burden were confirmed for a relatively short follow-up period of one month. Therefore, further studies are needed to confirm the long-term effects of this family psycho-educational intervention. Also more studies are recommended to perform and apply different models of psychoeducation, family to family intervention, etc.

## Conclusions

The present findings show the efficacy of a family psycho-educational intervention both in improving the patient’s clinical status and in reducing the family caregiver burden in an Iranian sample. These results suggest that even a short-term psycho-educational intervention for family members of patients with schizophrenia can improve the outcomes for patients and their families. In addition, our results showed a correlation between symptoms of schizophrenia and family burden. Further research on this approach is needed for family caregivers from culturally different backgrounds in the Iranian population as well as the populations of other countries. One of the differences of this study compared with other studies in Iran is that we have performed needs assessment before intervention. Longer follow-up periods are recommended to determine the long-term effects of family psycho educational intervention on outcomes for patients and their families.

## Competing interests

The authors declare that they have no competing interests.

## Authors’ contributions

FS, the main investigator, coordinated the research and wrote the first draft of the manuscript. MSH was responsible for data collection and contributed to the data analysis. AM assisted in the study design and liaised with the patients for the intervention. All the authors read and approved the final manuscript.

## Pre-publication history

The pre-publication history for this paper can be accessed here:

http://www.biomedcentral.com/1471-244X/12/48/prepub
